# Supplementary material for: Functional polymorphisms of the APOA1/C3/A4/A5-ZPR1-BUD13 gene cluster are associated with dyslipidemia in a sex-specific pattern
Source: PeerJ. 2019 Jan 4;6:e6175. doi: 10.7717/peerj.6175 (PMC6322487; doi:10.7717/peerj.6175)
Supplement: Supplemental Information 1 [file peerj-07-6175-s001.docx]

Table S1 Genotype and allele distributions and Hardy-Weinberg Equilibrium of the six SNPs

| SNPs | Total | Male | Female | *P*^a^ | *P*^b^ |
| --- | --- | --- | --- | --- | --- |
| *APOA1*-rs5072 |  |  |  |  |  |
| CC | 1779(46.3%) | 883(45.9%) | 896(46.8%) | 0.308 | 0.352 |
| TC | 1687(43.9%) | 840(43.7%) | 847(44.2%) |  |  |
| TT | 373(9.7%) | 201(10.4%) | 172(9.0%) |  |  |
| C | 5245(68.2%) | 2446(66.3%) | 2639(66.5%) | 0.842 |  |
| T | 2433(31.8%) | 1242(33.7%) | 1191(33.5%) |  |  |
| *APOA4*-rs5104 |  |  |  |  |  |
| AA | 1753(47.5%) | 885(48.1%) | 868(47.0%) | 0.179 | 0.573 |
| GA | 1570(42.5%) | 760(41.3%) | 810(43.8%) |  |  |
| GG | 367(10.0%) | 196(10.6%) | 171(9.2%) |  |  |
| A | 5076(68.8%) | 2530(68.7%) | 2546(68.8%) | 0.900 |  |
| G | 2304(31.2%) | 1152(31.3%) | 1152(31.2%) |  |  |
| *APOC3*-rs5128 |  |  |  |  |  |
| GG | 1819(47.4%) | 905(47.1%) | 914(47.7%) | 0.474 | 0.121 |
| GC | 1674(43.7%) | 833(43.4%) | 841(43.9%) |  |  |
| CC | 342(8.9%) | 182(9.5%) | 160(8.4%) |  |  |
| G | 5312(69.3%) | 2643(68.8%) | 2669(69.7%) | 0.415 |  |
| C | 2358(30.7%) | 1197(31.2%) | 1161(30.3%) |  |  |
| *APOA5*-rs651821 |  |  |  |  |  |
| TT | 1978(51.6%) | 959(49.9%) | 1019(53.3%) | 0.093 | 0.757 |
| CT | 1547(40.3%) | 799(41.5%) | 748(39.1%) |  |  |
| CC | 310(8.1%) | 165(8.6%) | 145(7.6%) |  |  |
| T | 5503(71.7%) | 2717(70.6%) | 2786(72.9%) | **0.032** |  |
| C | 2167(28.3%) | 1129(29.4%) | 1038(27.1%) |  |  |
| *ZPR1*-rs2075294 |  |  |  |  |  |
| GG | 2798(72.8%) | 1426(74.1%) | 1372(71.5%) | 0.203 | 0.889 |
| GT | 961(25.0%) | 458(23.8%) | 503(26.2%) |  |  |
| TT | 84(2.2%) | 41(2.1) | 43(2.3%) |  |  |
| G | 6557(85.3%) | 3310(86.0%) | 3247(84.6%) | 0.100 |  |
| T | 1129(14.7%) | 540(14.0%) | 589(15.4%) |  |  |
| *BUD13*-rs10488698 |  |  |  |  |  |
| CC | 3276(85.3%) | 1637(85.0%) | 1639(85.5%) | 0.919 | 0.759 |
| CT | 542(14.1%) | 276(14.4%) | 266(13.9%) |  |  |
| TT | 24(0.6%) | 12(0.6%) | 12(0.6%) |  |  |
| C | 7094(92.3%) | 3550(92.2%) | 3544(92.4%) | 0.707 |  |
| T | 590(7.7%) | 300(7.8%) | 290(7.6%) |  |  |

Note:

(a) *P* values were calculated between males and females, and *P* values no more than 0.05 were presented in bold.

(b) *P* values of Hardy-Weinberg Equilibrium in the overall group
