# Supplementary material for: Functional polymorphisms of the APOA1/C3/A4/A5-ZPR1-BUD13 gene cluster are associated with dyslipidemia in a sex-specific pattern
Source: PeerJ. 2019 Jan 4;6:e6175. doi: 10.7717/peerj.6175 (PMC6322487; doi:10.7717/peerj.6175)
Supplement: Supplemental Information 2 [file peerj-07-6175-s002.docx]

Table S2 Genotype distributions and allele frequencies in dyslipidemia and normal groups

| SNPs | | Hypertrigly-ceridemia | Normal | Hyperchol-esterolemia | Normal | High  LDL | Normal | Reduced  HDL | Normal |
| --- | --- | --- | --- | --- | --- | --- | --- | --- | --- |
| **Overall** |  |  |  |  |  |  |  |  |  |
| *APOA1*-rs5072 | CC | 456(41.2%) | 1323(48.4%) | 201(45.4%) | 1578(46.5%) | 175(49.9%) | 1604(46.0%) | 304(42.5%) | 1475(47.2%) |
|  | TC | 533(48.2%) | 1154(42.2%) | 192(43.3%) | 1495(44.0%) | 142(40.5%) | 1545(44.3%) | 324(45.3%) | 1363(43.6%) |
|  | TT | 117(10.6%) | 256(9.4%) | 50(11.3%) | 323(9.5%) | 34(9.7%) | 339(9.7%) | 87(12.2%) | 286(9.2%) |
| χ^2^ |  | 16.325 | | 1.414 | | 2.101 | | 8.583 | |
| *P* |  | **<0.001** | | 0.493 | | 0.350 | | **0.014** | |
|  | C | 1445(65.3%) | 3800(69.5%) | 594(67.0%) | 4651(68.5%) | 492(70.1%) | 4753(68.1%) | 932(65.2%) | 4313(69.0%) |
|  | T | 767(34.7%) | 1666(30.5%) | 292(33.0%) | 2141(31.5%) | 210(29.9%) | 2223(31.9%) | 498(34.8%) | 1935(31.0%) |
| χ^2^ |  | 12.803 | | 0.745 | | 1.123 | | 7.990 | |
| *P* |  | **<0.001** | | 0.388 | | 0.289 | | **0.005** | |
| *APOA4*-rs5104 | AA | 462(43.5%) | 1291(49.1%) | 196(46.2%) | 1557(47.7%) | 172(50.9%) | 1581(47.2%) | 296(42.7%) | 1457(48.6%) |
|  | GA | 482(45.4%) | 1088(41.4%) | 181(42.7%) | 1389(42.5%) | 137(40.5%) | 1433(42.8%) | 315(45.5%) | 1255(41.9%) |
|  | GG | 117(11.0%) | 250(9.5%) | 47(11.1%) | 320(9.8%) | 29(8.6%) | 338(10.1%) | 82(11.8%) | 285(9.5%) |
| χ^2^ |  | 9.580 | | 0.793 | | 1.948 | | 8.877 | |
| *P* |  | **0.008** | | 0.673 | | 0.378 | | **0.012** | |
|  | A | 1406(66.3%) | 3670(69.8%) | 573(67.6%) | 4503(68.9%) | 481(71.2%) | 4595(68.5%) | 907(65.4%) | 4169(69.6%) |
|  | G | 716(33.7%) | 1588(30.2%) | 275(32.4%) | 2029(31.1%) | 195(28.8%) | 2109(31.5%) | 479(34.6%) | 1825(30.4%) |
| χ^2^ |  | 8.824 | | 0.653 | | 1.952 | | 8.868 | |
| *P* |  | **0.003** | | 0.419 | | 0.162 | | **0.003** | |
| *APOC3*-rs5128 | GG | 466(42.3%) | 1353(49.5%) | 205(46.4%) | 1614(47.6%) | 178(50.7%) | 1641(47.1%) | 310(43.4%) | 1509(48.4%) |
|  | GC | 532(48.3%) | 1142(41.8%) | 192(43.4%) | 1482(43.7%) | 141(40.2%) | 1533(44.0%) | 328(45.9%) | 1346(43.1%) |
|  | CC | 103(9.4%) | 239(8.7%) | 45(10.2%) | 297(8.8%) | 32(9.1) | 310(8.9%) | 77(10.8%) | 265(8.5%) |
| χ^2^ |  | 16.534 | | 1.015 | | 1.966 | | 7.450 | |
| *P* |  | **<0.001** | | 0.602 | | 0.374 | | **0.024** | |
|  | G | 1464(66.5%) | 3848(70.4%) | 602(68.1%) | 4710(69.4%) | 497(70.8%) | 4815(69.1%) | 948(66.3%) | 4364(69.9%) |
|  | C | 738(33.5%) | 1620(29.6%) | 282(31.9%) | 2076(30.6%) | 205(29.2%) | 2153(30.9%) | 482(33.7%) | 1876(30.1%) |
| χ^2^ |  | 11.146 | | 0.629 | | 0.862 | | 7.248 | |
| *P* |  | **0.001** | | 0.428 | | 0.353 | | **0.007** | |
| *APOA5*-rs651821 | TT | 440(39.8%) | 1538(56.4%) | 203(45.9%) | 1775(52.3%) | 178(50.7%) | 1800(51.7%) | 272(38.0%) | 1706(54.7%) |
|  | CT | 517(46.7%) | 1030(37.7%) | 199(45.0%) | 1348(39.7%) | 152(43.3%) | 1395(40.0%) | 335(46.8%) | 1212(38.9%) |
|  | CC | 149(13.5%) | 161(5.9%) | 40(9.0%) | 270(8.0%) | 21(6.0%) | 289(8.3%) | 109(15.2%) | 201(6.4%) |
| χ^2^ |  | 113.561 | | 6.386 | | 3.008 | | 96.116 | |
| *P* |  | **<0.001** | | **0.041** | | 0.222 | | **<0.001** | |
|  | T | 1397(63.2%) | 4106(75.2%) | 605(68.4%) | 4898(72.2%) | 508(72.4%) | 4995(71.7%) | 879(61.4%) | 4624(74.1%) |
|  | C | 815(36.8%) | 1352(24.8%) | 279(31.6%) | 1888(27.8%) | 194(27.6%) | 1973(28.3%) | 553(38.6%) | 1614(25.9%) |
| χ^2^ |  | 113.194 | | 5.394 | | 0.145 | | 93.307 | |
| *P* |  | **<0.001** | | **0.020** | | 0.703 | | **<0.001** | |
| *ZPR1*-rs2075294 | GG | 828(74.8%) | 1970(72.0%) | 336(76.0%) | 2462(72.4%) | 264(75.2%) | 2534(72.6%) | 537(74.9%) | 2261(72.3%) |
|  | GT | 257(23.2%) | 704(25.7%) | 95(21.5%) | 866(25.5%) | 80(22.8%) | 881(25.2%) | 163(22.7%) | 798(25.5%) |
|  | TT | 22(2.0%) | 62(2.3%) | 11(2.5%) | 73(2.1%) | 7(2.0%) | 77(2.2%) | 17(2.4%) | 67(2.1%) |
| χ^2^ |  | 3.119 | | 3.382 | | 1.129 | | 2.487 | |
| *P* |  | 0.210 | | 0.184 | | 0.569 | | 0.288 | |
|  | G | 1913(86.4%) | 4644(84.9%) | 767(86.8%) | 5790(85.1%) | 608(86.6%) | 5949(85.2%) | 1237(86.3%) | 5320(85.1%) |
|  | T | 301(13.6%) | 828(15.1%) | 117(13.2%) | 1012(14.9%) | 94(13.4%) | 1035(14.8%) | 197(13.7%) | 932(14.9%) |
| χ^2^ |  | 2.969 | | 1.685 | | 1.040 | | 1.273 | |
| *P* |  | 0.085 | | 0.194 | | 0.308 | | 0.259 | |
| *BUD13*-rs10488698 | CC | 967(87.2%) | 2309(84.5%) | 377(84.9%) | 2899(85.3%) | 300(85.2%) | 2976(85.3%) | 634(88.2%) | 2642(84.6%) |
|  | CT | 136(12.3%) | 406(14.9%) | 64(14.4%) | 478(14.1%) | 49(13.9%) | 493(14.1%) | 82(11.4%) | 460(14.7%) |
|  | TT | 6(0.5%) | 18(0.7%) | 3(0.7%) | 21(0.6%) | 3(0.9%) | 21(0.6%) | 3(0.4%) | 21(0.7%) |
| χ^2^ |  | 4.611 | | 0.062 | | 0.331 | | 6.067 | |
| *P* |  | 0.100 | | 0.969 | | 0.847 | | **0.048** | |
|  | C | 2070(93.3%) | 5024(91.9%) | 818(92.1%) | 6276(92.3%) | 649(92.2%) | 6445(92.3%) | 1350(93.9%) | 5744(92.0%) |
|  | T | 148(6.7%) | 442(8.1%) | 70(7.9%) | 520(7.7%) | 55(7.8%) | 535(7.7%) | 88(6.1%) | 502(8.0%) |
| χ^2^ |  | 4.448 | | 0.059 | | 0.020 | | 6.063 | |
| *P* |  | **0.035** | | 0.808 | | 0.888 | | **0.014** | |
| **Male** |  |  |  |  |  |  |  |  |  |
| *APOA1*-rs5072 | CC | 269(40.6%) | 614(48.6%) | 88(41.1%) | 795(46.5%) | 71(49.0%) | 812(45.6%) | 184(41.0%) | 699(47.4%) |
|  | TC | 313(47.3%) | 527(41.8%) | 97(45.3%) | 743(43.5%) | 60(41.4%) | 780(43.8%) | 205(45.6%) | 635(43.1%) |
|  | TT | 80(12.1%) | 121(9.6%) | 29(13.6%) | 172(10.1%) | 14(9.6%) | 187(10.5%) | 60(13.4%) | 141(9.6%) |
| χ^2^ |  | 11.707 | | 3.570 | | 0.603 | | 8.384 | |
| *P* |  | **0.003** | | 0.168 | | 0.740 | | **0.015** | |
|  | C | 851(64.3%) | 1759(69.6%) | 273(63.8%) | 2333(68.2%) | 202(69.7%) | 2404(67.6%) | 573(63.8%) | 2033(68.9%) |
|  | T | 473(35.7%) | 769(30.4%) | 155(36.2%) | 1087(31.8%) | 88(30.3%) | 1154(32.4%) | 325(36.2%) | 917(31.1%) |
| χ^2^ |  | 11.197 | | 3.417 | | 0.535 | | 8.214 | |
| *P* |  | **0.001** | | 0.065 | | 0.464 | | **0.004** | |
| *APOA4*-rs5104 | AA | 274(43.3%) | 611(50.6%) | 89(43.4%) | 796(48.7%) | 73(52.1%) | 812(47.7%) | 182(42.0%) | 703(49.9%) |
|  | GA | 286(45.2%) | 474(39.2%) | 90(43.9%) | 670(40.9%) | 55(39.3%) | 705(41.4%) | 197(45.5%) | 563(40.0%) |
|  | GG | 73(11.5%) | 123(10.2%) | 26(12.7%) | 170(10.4%) | 12(8.6%) | 184(10.8%) | 54(12.5%) | 141(10.1%) |
| χ^2^ |  | 8.861 | | 2.323 | | 1.281 | | 8.502 | |
| *P* |  | **0.012** | | 0.313 | | 0.527 | | **0.014** | |
|  | A | 834(65.9%) | 1696(70.2%) | 268(65.4%) | 2262(69.1%) | 201(71.8%) | 2329(68.5%) | 561(64.8%) | 1969(70.0%) |
|  | G | 432(34.1%) | 720(29.8%) | 142(34.6%) | 1010(30.9%) | 79(28.2%) | 1073(31.5%) | 305(35.2%) | 845(30.0%) |
| χ^2^ |  | 7.218 | | 2.404 | | 1.331 | | 8.306 | |
| *P* |  | **0.007** | | 0.121 | | 0.249 | | **0.004** | |
| *APOC3*-rs5128 | GG | 276(41.8%) | 629(49.9%) | 88(41.3%) | 817(47.9%) | 71(49.0%) | 834(47.0%) | 189(42.3%) | 716(48.6%) |
|  | GC | 313(47.4%) | 520(41.3%) | 99(46.5%) | 734(43.0%) | 60(41.4%) | 773(43.5%) | 206(46.1%) | 627(42.6%) |
|  | CC | 71(10.8) | 111(8.8%) | 26(12.2%) | 156(9.1%) | 14(9.6%) | 168(9.5%) | 52(11.6%) | 130(8.8%) |
| χ^2^ |  | 11.548 | | 4.131 | | 0.262 | | 6.742 | |
| *P* |  | **0.003** | | 0.127 | | 0.877 | | **0.034** | |
|  | G | 865(65.5%) | 1778(70.6%) | 275(64.6%) | 2368(69.4%) | 202(69.7%) | 2441(68.8%) | 584(65.3%) | 2059(69.9%) |
|  | C | 455(34.5%) | 742(29.4%) | 151(35.4%) | 1046(30.6%) | 88(30.3%) | 1109(31.2%) | 310(34.7%) | 887(30.1%) |
| χ^2^ |  | 10.196 | | 4.080 | | 0.100 | | 6.668 | |
| *P* |  | **0.001** | | **0.043** | | 0.752 | | **0.010** | |
| *APOA5*-rs651821 | TT | 259(39.2%) | 700(55.5%) | 93(43.7%) | 866(50.6%) | 76(52.8%) | 883(49.6%) | 172(38.4%) | 787(53.4%) |
|  | CT | 313(47.3%) | 486(38.5%) | 97(45.5%) | 702(41.1%) | 60(41.7%) | 739(41.5%) | 211(47.1%) | 588(39.9%) |
|  | CC | 89(13.5%) | 76(6.0%) | 23(10.8%) | 142(8.3%) | 8(5.6%) | 157(8.8%) | 65(14.5%) | 100(6.8%) |
| χ^2^ |  | 59.231 | | 4.142 | | 1.924 | | 43.681 | |
| *P* |  | **<0.001** | | 0.126 | | 0.382 | | **<0.001** | |
|  | T | 813(62.3%) | 1886(74.7%) | 283(66.4%) | 2434(71.2%) | 212(73.6%) | 2505(70.4%) | 555(61.9%) | 2164(73.3%) |
|  | C | 491(37.7%) | 638(25.3%) | 143(33.6%) | 986(28.8%) | 76(26.4%) | 1053(29.6%) | 341(38.1%) | 788(26.7%) |
| χ^2^ |  | 63.329 | | 4.100 | | 1.321 | | 42.819 | |
| *P* |  | **<0.001** | | **0.043** | | 0.250 | | **<0.001** | |
| *ZPR1*-rs2075294 | GG | 497(75.0%) | 829(73.6%) | 171(80.3%) | 1255(73.3%) | 119(82.1%) | 1307(73.4%) | 335(74.4%) | 1091(74.0%) |
|  | GT | 155(23.4%) | 303(24.0%) | 36(16.9%) | 422(24.7%) | 24(16.5%) | 434(24.4%) | 107(23.8%) | 351(23.8%) |
|  | TT | 11(1.6%) | 30(2.4%) | 6(2.8%) | 35(2.0%) | 2(1.4%) | 39(2.2%) | 8(1.8%) | 33(2.2%) |
| χ^2^ |  | 1.232 | | 6.555 | | 5.222 | | 0.353 | |
| *P* |  | 0.540 | | **0.038** | | 0.073 | | 0.838 | |
|  | G | 1149(86.7%) | 1961(84.4%) | 378(88.7%) | 2932(85.6%) | 262(90.3%) | 3048(85.6%) | 777(86.3%) | 2533(85.9%) |
|  | T | 177(13.3%) | 363(15.6%) | 48(11.3%) | 492(14.4%) | 28(9.7%) | 512(14.4%) | 123(13.7%) | 417(14.1%) |
| χ^2^ |  | 3.455 | | 3.022 | | 4.969 | | 0.126 | |
| *P* |  | 0.063 | | 0.082 | | **0.026** | | 0.723 | |
| *BUD13*-rs10488698 | CC | 575(86.6%) | 1062(84.2%) | 178(83.2%) | 1459(85.3%) | 114(78.6%) | 1523(85.6%) | 396(88.0%) | 1241(84.1%) |
|  | CT | 86(12.9%) | 190(15.1%) | 33(1.4%) | 243(14.2%) | 28(19.3%) | 248(13.9%) | 52(11.6%) | 224(15.2%) |
|  | TT | 3(0.5%) | 9(0.7%) | 3(1.4%) | 9(0.5%) | 3(2.1%) | 9(0.5%) | 2(0.4%) | 10(0.7%) |
| χ^2^ |  | 2.126 | | 2.636 | | 8.721 | | 4.078 | |
| *P* |  | 0.345 | | 0.268 | | **0.013** | | 0.130 | |
|  | C | 1236(93.1%) | 2314(91.8%) | 389(90.9%) | 3161(92.4%) | 256(88.3%) | 3294(92.5%) | 844(93.8%) | 2706(91.7%) |
|  | T | 92(6.9%) | 208(8.2%) | 39(9.1%) | 261(7.6%) | 34(11.7%) | 266(7.5%) | 56(6.2%) | 244(8.3%) |
| χ^2^ |  | 2.109 | | 1.168 | | 6.748 | | 4.029 | |
| *P* |  | 0.146 | | 0.280 | | **0.009** | | **0.045** | |
| **Female** |  |  |  |  |  |  |  |  |  |
| *APOA1*-rs5072 | CC | 187(42.1%) | 709(48.2%) | 113(49.3%) | 783(46.4%) | 104(50.5%) | 792(46.3%) | 120(45.1%) | 776(47.1) |
|  | TC | 220(49.6%%) | 627(42.6%) | 95(41.5%) | 752(44.6%) | 82(39.8%) | 765(44.8%) | 119(44.7%) | 728(44.1%) |
|  | TT | 37(8.3%) | 135(9.2%) | 21(9.2%) | 151(9.0%) | 20(9.7%) | 152(8.9%) | 27(10.2%) | 145(8.8%) |
| χ^2^ |  | 6.665 | | 0.817 | | 1.832 | | 0.673 | |
| *P* |  | **0.036** | | 0.665 | | 0.400 | | 0.714 | |
|  | C | 594(66.9%) | 2085(69.9%) | 321(70.1%) | 2318(68.7%) | 290(70.4%) | 2349(68.7%) | 359(67.5%) | 2280(69.1%) |
|  | T | 294(33.1%) | 897(30.1%) | 137(29.9%) | 1054(31.3%) | 122(29.6%) | 1069(31.3%) | 173(32.5%) | 1018(30.9%) |
| χ^2^ |  | 2.944 | | 0.340 | | 0.475 | | 0.583 | |
| *P* |  | 0.086 | | 0.560 | | 0.491 | | 0.445 | |
| *APOA4*-rs5104 | AA | 188(43.9%) | 680(47.9%) | 107(48.9%) | 761(46.7%) | 99(50.0%) | 769(46.6%) | 114(43.8%) | 754(47.5%) |
|  | GA | 196(45.8%) | 614(43.2%) | 91(41.6%) | 719(44.1%) | 82(41.4%) | 728(44.1%) | 118(45.4%) | 692(43.5%) |
|  | GG | 44(10.3%) | 127(8.9%) | 21(9.6%) | 150(9.2%) | 17(8.6%) | 154(9.3%) | 28(10.8%) | 143(9.0%) |
| χ^2^ |  | 2.225 | | 0.513 | | 0.836 | | 1.547 | |
| *P* |  | 0.329 | | 0.774 | | 0.658 | | 0.461 | |
|  | A | 572(66.8%) | 1974(69.5%) | 305(69.6%) | 2241(68.7%) | 280(70.7%) | 2266(68.6%) | 346(66.5%) | 2200(62.4%) |
|  | G | 284(33.2%) | 868(30.5%) | 133(30.4%) | 1019(31.3%) | 116(29.3%) | 1036(31.4%) | 174(33.5%) | 978(37.6%) |
| χ^2^ |  | 2.131 | | 0.143 | | 0.715 | | 1.505 | |
| *P* |  | 0.144 | | 0.705 | | 0.398 | | 0.220 | |
| *APOC3*-rs5128 | GG | 190(43.1%) | 724(49.1%) | 117(51.1%) | 797(47.3%) | 107(52.0%) | 807(47.2%) | 121(45.2%) | 793(48.1%) |
|  | GC | 219(49.7%) | 622(42.2%) | 93(40.6%) | 748(44.4%) | 81(39.3%) | 760(44.5%) | 122(45.5%) | 719(43.7%) |
|  | CC | 32(7.2%) | 128(8.7%) | 19(8.3%) | 141(8.4%) | 18(8.7%) | 142(8.3%) | 25(9.3%) | 135(8.2%) |
| χ^2^ |  | 7.721 | | 1.264 | | 2.009 | | 0.971 | |
| *P* |  | **0.021** | | 0.531 | | 0.366 | | 0.616 | |
|  | G | 599(67.9%) | 2070(70.2%) | 327(71.4%) | 2342(69.5%) | 295(71.6%) | 2374(69.5%) | 364(67.9%) | 2305(70.0%) |
|  | C | 283(32.1%) | 878(29.8%) | 131(28.6%) | 1030(30.5%) | 117(28.4%) | 1044(30.5%) | 172(32.1%) | 989(30.0%) |
| χ^2^ |  | 1.705 | | 0.721 | | 0.802 | | 0.931 | |
| *P* |  | 0.192 | | 0.396 | | 0.371 | | 0.335 | |
| *APOA5*-rs651821 | TT | 181(40.7%) | 838(57.1%) | 110(48.0%) | 909(54.0%) | 102(49.3%) | 917(53.8%) | 100(37.3%) | 919(55.9%) |
|  | CT | 204(45.8%) | 544(37.1%) | 102(44.6%) | 646(38.4%) | 92(44.4%) | 656(38.5%) | 124(46.3%) | 624(38.0%) |
|  | CC | 60(13.5%) | 85(5.8%) | 17(7.4%) | 128(7.6%) | 13(6.3%) | 132(7.7%) | 44(16.4%) | 101(6.1%) |
| χ^2^ |  | 50.649 | | 3.313 | | 2.905 | | 51.083 | |
| *P* |  | **<0.001** | | 0.191 | | 0.234 | | **<0.001** | |
|  | T | 566(63.6%) | 2220(75.7%) | 322(70.3%) | 2464(73.2%) | 296(71.5%) | 2490(73.0%) | 324(60.4%) | 2462(74.9%) |
|  | C | 324(36.4%) | 714(24.3%) | 136(29.7%) | 902(26.8%) | 118(28.5%) | 920(27.0%) | 212(39.6%) | 826(25.1%) |
| χ^2^ |  | 50.297 | | 1.711 | | 0.433 | | 48.529 | |
| *P* |  | **<0.001** | | 0.191 | | 0.511 | | **<0.001** | |
| *ZPR1*-rs2075294 | GG | 331(74.5%) | 1041(70.6%) | 165(72.0%) | 1207(71.5%) | 145(70.4%) | 1227(71.7%) | 202(75.6%) | 1170(70.9%) |
|  | GT | 102(23.0%) | 401(27.2%) | 59(25.8%) | 444(26.3%) | 56(27.2%) | 447(26.1%) | 56(21.0%) | 447(27.1%) |
|  | TT | 11(2.5%) | 32(2.2%) | 5(2.2%) | 38(2.2%) | 5(2.4%) | 38(2.2%) | 9(3.4%) | 34(2.0%) |
| χ^2^ |  | 3.208 | | 0.035 | | 0.159 | | 5.762 | |
| *P* |  | 0.201 | | 0.983 | | 0.924 | | 0.056 | |
|  | G | 764(86.0%) | 2483(84.7%) | 389(84.9%) | 2858(84.6%) | 346(84.0%) | 2901(84.7%) | 460(86.1%) | 2787(84.4%) |
|  | T | 124(14.0%) | 465(15.3%) | 69(15.1%) | 520(15.4%) | 66(16.0%) | 523(15.3%) | 74(13.9%) | 515(15.6%) |
| χ^2^ |  | 0.902 | | 0.033 | | 0.157 | | 1.069 | |
| *P* |  | 0.342 | | 0.855 | | 0.692 | | 0.301 | |
| *BUD13*-rs10488698 | CC | 392(88.1%) | 1247(84.7%) | 199(86.5%) | 1440(85.4%) | 186(89.9%) | 1453(85.0%) | 238(88.5%) | 1401(85.0%) |
|  | CT | 50(11.2%) | 216(14.7%) | 31(13.5%) | 235(13.9%) | 21(10.1%) | 245(14.3%) | 30(11.2%) | 236(14.3%) |
|  | TT | 3(0.7%) | 9(0.6%) | 0(0.0%) | 12(0.7%) | 0(0.0%) | 12(0.7%) | 1(0.3%) | 11(0.7%) |
| χ^2^ |  | 3.387 | | 1..698 | | 4.296 | | 2.320 | |
| *P* |  | 0.184 | | 0.428 | | 0.117 | | 0.313 | |
|  | C | 834(93.7%) | 2710(92.1%) | 429(93.3%) | 3115(92.3%) | 393(94.9%) | 3151(92.1%) | 506(94.1%) | 3038(92.2%) |
|  | T | 56(6.3%) | 234(7.9%) | 31(6.7%) | 259(7.7%) | 21(5.1%) | 269(7.9%) | 32(5.9%) | 258(7.8%) |
| χ^2^ |  | 2.681 | | 0.509 | | 4.120 | | 2.337 | |
| *P* |  | 0.102 | | 0.476 | | **0.042** | | 0.126 | |

Note:

*P* values in bold indicated that differences were significant compared to the reference group (*P*<0.05).
