# Supplementary material for: Functional polymorphisms of the APOA1/C3/A4/A5-ZPR1-BUD13 gene cluster are associated with dyslipidemia in a sex-specific pattern
Source: PeerJ. 2019 Jan 4;6:e6175. doi: 10.7717/peerj.6175 (PMC6322487; doi:10.7717/peerj.6175)
Supplement: Supplemental Information 3 [file peerj-07-6175-s003.docx]

Table S3 Association between the number of risk alleles and dyslipidemia

| Variables | *P*^a^ | OR (95% CI) |
| --- | --- | --- |
| Number of risk alleles | **<0.001** | 1.073(1.035-1.113) |
| Gender (Female/Male) | 0.651 | 1.039(0.880-1.228) |
| Age | **<0.001** | 1.017(1.008-1.026) |
| BMI | **0.001** | 1.066(1.025-1.109) |
| Waist circumference | **<0.001** | 1.112(1.095-1.128) |

Note:

BMI, body mass index

OR, odds ratio

CI, confidence interval

(a)*P* values of no more than 0.05 were presented in bold.
