# Supplementary material for: Functional polymorphisms of the APOA1/C3/A4/A5-ZPR1-BUD13 gene cluster are associated with dyslipidemia in a sex-specific pattern
Source: PeerJ. 2019 Jan 4;6:e6175. doi: 10.7717/peerj.6175 (PMC6322487; doi:10.7717/peerj.6175)
Supplement: Supplemental Information 4 [file peerj-07-6175-s004.docx]

Table S4 Association between haplotypes in blocks and different types of dyslipidemia stratified by genders

| Haplotypes | | ORs^a^ | | | |
| --- | --- | --- | --- | --- | --- |
|  |  | Hypertrigly-ceridemia | Hypercholes-terolemia | High LDL | Reduced HDL |
| **Overall** |  |  |  |  |  |
| Block1 | CGT | **0.70(0.63-0.77)** | 0.91(0.79-1.05) | 1.08(0.93-1.26) | **0.68(0.61-0.76)** |
|  | CGC | **1.76(1.59-1.96)** | **1.19(1.03-1.34)** | 0.97(0.82-1.15) | **1.80(1.59-2.03)** |
|  | CTT | 0.88(0.77-1.02) | 0.88(0.72-1.08) | 0.90(0.71-1.12) | 0.91(0.77-1.07) |
|  | TGT | **0.81(0.67-0.99)** | 1.03(0.80-1.34) | 1.02(0.76-1.36) | **0.75(0.59-0.94)** |
| Block2 | AGC | **0.84(0.76-0.93)** | 0.94(0.81-1.08) | 1.06(0.90-1.25) | **0.86(0.76-0.96)** |
|  | GCT | **1.22(1.09-1.36)** | 1.09(0.93-1.27) | 0.87(0.73-1.04) | **1.22(1.08-1.39)** |
|  | GGC | 0.98(0.78-1.24) | 1.06(0.77-1.47) | 1.17(0.83-1.66) | 0.97(0.74-1.28) |
|  | ACT | 1.09(0.85-1.38) | **0.70(0.49-0.99)** | 1.23(0.86-1.75) | 0.97(0.73-1.29) |
| **Male** |  |  |  |  |  |
| Block1 | CGT | **0.68(0.60-0.78)** | 0.88(0.72-1.08) | 1.15(0.91-1.46) | **0.70(0.61-0.82)** |
|  | CGC | **1.74(1.51-2.01)** | **1.25(1.01-1.55)** | 0.86(0.66-1.12) | **1.68(1.44-1.97)** |
|  | CTT | 0.92(0.76-1.11) | 0.75(0.55-1.03) | **0.64(0.43-0.95)** | 0.96(0.77-1.19) |
|  | TGT | 0.83(0.64-1.07) | 1.22(0.86-1.73) | **1.65(1.12-2.41)** | **0.74(0.55-1.00)** |
| Block2 | AGC | **0.82(0.72-0.94)** | 0.84(0.69-1.03) | 1.10(0.85-1.41) | **0.83(0.71-0.97)** |
|  | GCT | **1.30(1.12-1.50)** | **1.25(1.01-1.56)** | 0.88(0.67-1.16) | **1.30(1.11-1.54)** |
|  | GGC | 1.02(0.73-1.41) | 0.86(0.51-1.45) | 0.97(0.53-1.76) | 0.88(0.60-1.30) |
|  | ACT | 0.83(0.59-1.17) | 1.05(0.64-1.71) | 1.35(0.79-2.29) | 0.88(0.60-1.29) |
| **Female** |  |  |  |  |  |
| Block1 | CGT | **0.71(0.61-0.83)** | 1.04(0.85-1.27) | 1.02(0.83-1.25) | **0.65(0.54-0.78)** |
|  | CGC | **1.77(1.51-2.07)** | 1.14(0.92-1.42) | 1.07(0.86-1.35) | **1.95(1.61-2.36)** |
|  | CTT | 0.87(0.70-1.08) | 0.98(0.75-1.29) | 1.07(0.81-1.41) | 0.88(0.67-1.14) |
|  | TGT | 0.78(0.57-1.05) | 0.87(0.59-1.28) | **0.63(0.40-0.99)** | 0.74(0.51-1.09) |
| Block2 | AGC | **0.85(0.73-0.99)** | 1.03(0.85-1.27) | 1.02(0.83-1.26) | 0.90(0.74-1.08) |
|  | GCT | 1.11(0.94-1.32) | 0.96(0.76-1.20) | 0.87(0.69-1.11) | 1.09(0.89-1.34) |
|  | GGC | 1.21(0.88-1.67) | 1.22(0.81-1.84) | 1.31(0.86-2.00) | 1.19(0.81-1.76) |
|  | ACT | 1.19(0.83-1.70) | 0.84(0.50-1.40) | 1.09(0.67-1.78) | 1.10(0.71-1.70) |

Note:

OR, odds ratio

SNPs in block 1 were as follows: rs10488698, rs2075294, rs651821; SNPs in block 2 were as follows: rs5104, rs5128, rs5072.

(a) *P* values of less than 0.05 were considered to be significant and were presented in bold.
